# Perceptions and Preventive Practices Regarding COVID-19 Pandemic Outbreak and Oral Health Care Perceptions during the Lockdown: A Cross-Sectional Survey from Saudi Arabia

**DOI:** 10.3390/healthcare9080959

**Published:** 2021-07-29

**Authors:** Abdullah Alassaf, Basim Almulhim, Sara Ayid Alghamdi, Sreekanth Kumar Mallineni

**Affiliations:** Department of Preventive Dental Science, College of Dentistry, Majmaah University, Al-Majmaah 11952, Saudi Arabia; am.assaf@mu.edu.sa (A.A.); b.almulhim@mu.edu.sa (B.A.); Sa.mohammed@mu.edu.sa (S.A.A.)

**Keywords:** Coronavirus, Saudi Arabia, perception, COVID-19, prevention

## Abstract

Aims: The study aimed to evaluate perceptions and preventive practices regarding the COVID-19 pandemic and oral health care perceptions during the lockdown in the Saudi Arabian population. Materials and Method: This cross-sectional study was performed by collecting the data from individuals belonging to various parts of the Saudi Arabian Population through an online self-reported questionnaire. The questionnaire had two main parts: first comprised of demographic data include the region of residence, gender, nationality, age, the number of family members, monthly income of the family, and the second was further divided into three sections of perception (P), practice (PRA) and oral health care practice (D) questions. All these (P, PRA, and D) were analyzed by comparing all of the demographic characteristics. Statistical analysis was performed using SPSS IBM (version 21.0), and statistical significance was set at a 5% level. Results: Overall, 2013 participants (54% males and 46% females) contributed to the Saudi Arabia study. Only 5% of non-Saudis live in Saudi Arabia were participated in the study, while the majority of participants were of 21–40 years age group (45%), 59% of having more than five family members, and 60% of them had ≤10 K Suadi riyal monthly income respectively. The majority of the participants were from Riyadh (33.7%) and Asir (25.1%) in the study. Overall, 89.5% of the participants were aware of the COVID-19 global pandemic. The majority of the participants (55%) from Saudi Arabia utilized the Ministry of Health website, a source of information regarding COVID-19. However, 56.5% of the participants had COVID-19 related perception, and 74.3% followed an appropriate preventive practice. Approximately 60% had good oral health practice. The study participants showed mixed opinions on perceptions regarding COVID-19, preventive practice, and oral health practices. Conclusion: The present study suggested that the Saudi Arabian population has good attention to COVID-19, but preventive practice and oral health perception need better awareness to control this novel virus spread. The Ministry of Health website utilized as a significant source of information among the Saudi Arabian population regarding COVID-19.

## 1. Introduction

Coronavirus disease is referred to as “COVID-19” and is caused by a novel respiratory virus [1] and this single-stranded RNA virus belongs to a Coronaviridae family [2,3]. It was transmitted initially from animal-to-human and then human-to-human [1,4]. The governments and public health organizations have adopted numerous measures worldwide to improve awareness, raise knowledge, and increase preventive practice to control COVID-19 transmission [5,6]. It originated from China at the end of the year 2019 and subsequently circulated globally [7]. The first positive case reported of COVID-19 was by the Ministry of Health (MOH), Kingdom of Saudi Arabia, on 2nd March 2020. Eventually, the number of positive cases was accelerated in a month, and it has become a big challenge for healthcare professionals [7]. As of 31 December 2020, 362,714 cases were registered in Saudi Arabia [8]. The government authorities of Saudi Arabia have focused on precautionary measures with general population interest [9]. These include lockdown, airport and border surveillance, quarantine of suspicious and infected patients, and infection control training for healthcare workers [10]. In addition, public places are at potential risk of spreading COVID-19 to family and friends, and colleagues [11]. Therefore, it became essential that the public know the disease and preventive practices suggested by health authorities. COVID-19 transmits through air droplets, contaminated surfaces, mucus membranes and secretions from the nose or mouth, or eyes, and close contact with infected persons [12,13]. COVID-19 cases are usually symptomatic; however, recently, asymptomatic patients have also been reported, which has become a significant concern for health care professionals. Dry cough, fatigue, fever, dyspnea, and myalgia, commonly reported symptoms in COVID-19 positive individuals. Subsequently, an Italian study reported that there will be an alteration in smell and taste in individuals with COVID-19 [14]. Additional observed symptoms include abdominal pain, headache, diarrhea, and sore throat. The severe stage of COVID-19 is characterized by septic shock, acute respiratory distress syndrome, bleeding, coagulation disorders, and metabolic acidosis [15,16].

The world health organization (WHO) recommends specific preventive personal hygiene measures, including using face masks, repeated handwashing with water and soap, using hand sanitizers, avoiding touching mouth, eyes, and nose frequently, cleanliness, and social distancing well as careful handling of purchased products. These measures are very strictly acclaimed to control the spread of COVID-19 disease and to protect the people from this pandemic. Nevertheless, a lack of understanding of the COVID-19 risk factors among people has been observed worldwide [10,17]. People’s understanding and adherence to preventive measures play an essential part in controlling the contraction of COVID-19 [9]. Di Lorenzo and Di Trolio [16] opined that strict obedience to the rules proposed by health authorities might be useful in avoiding the transmission of COVID-19. This tractability depends on their awareness, perception, and preventive practice factors. Hence, there is a need for a survey to check for the perception, preventive practice, and oral health care perceptions in Saudi Arabia. Oral health care professionals are at potential risk of acquiring COVID-19 because most dental procedures are aerosol generated [5,13,18]. It also impacted the people whether to seek dental treatment during this COVID-19 pandemic or not. Nonetheless, there is no data available on the oral health care perceptions of the Saudi Arabian population. However, lack of awareness and inadequate understanding of the people at risk has led to the COVID-19 pandemic outbreak of this disease, resulting in colossal morbidity and mortality worldwide. Henceforth, the study was aimed to evaluate perceptions and preventive practices regarding the COVID-19 pandemic and oral health care perceptions during the lockdown: a population-based cross-sectional survey from Saudi Arabia.

## 2. Materials and Method

The Ethical Committee Clearance was obtained from Majmaah University, Al-Majmaah, Saudi Arabia, under IRB No: MUREC-June-10/Com-2020/32-3. This cross-sectional survey was conducted from 1 June 2020 to 31 July 2020 among the people from Saudi Arabia, and the self-administered questionnaire was sent through digital platforms online via google forms. The questionnaire comprised two main parts: first part consisted of demographic data including the region of residence (Riyadh, Al Baha, Makkah, Qassim, Northern Border region, Tabuk, Jazan, Asir, Hail, Madinah, Najran, Eastern, and Al Jouf), gender (male and female), nationality (Saudi and Non-Saudi), age in years (≤20, 21–40, 41–60, >60), number of family members (≤5 and >5), and monthly income of the family (≤10 KSAR and >10 K Saudi riyal (SAR). The second part had three sections as follows: Six questions for perception (P), preventive practice (PRA), and three questions for oral health care perception (D), shown in Table 1. A pilot survey was conducted among the team members that filled and reviewed all the questions. The changes were made accordingly prior to the distribution of the questionnaire among the participants. The responses obtained from the pilot study were not included in the final data analysis. The validation of the questionnaire was completed and translated into Arabic by a native speaker (AA) and edited prior to distribution. The translations were made accessible in English and Arabic languages. The participants made it an easy and understandable form. The effect of age, gender, nationality, number of family members, and monthly income was considered for evaluating P, PRA, and D regarding COVID-19. The questionnaire was sent as a link via social media to the Saudi Arabian population using Google form. The recruitment and consent to participate in the study followed the participants’ willingness to complete the questionnaire. In the perception of the feasibility of analysis “yes” as a positive response and “no” as a negative response. Similarly, we followed the same criteria for all the domains. The mean percentages of the positive responses for all the questions were used for the measurement. The Chi-square tests were used for comparisons of percentages. All the demographic characteristics of participants were presented using summary statistics. The statistical analysis was performed using IBM SPSS Statistics (Version 21.0. Armonk, NY, USA: IBM Corp); statistical significance was set at a 5% level.

## 3. Results

**Figure 1:**
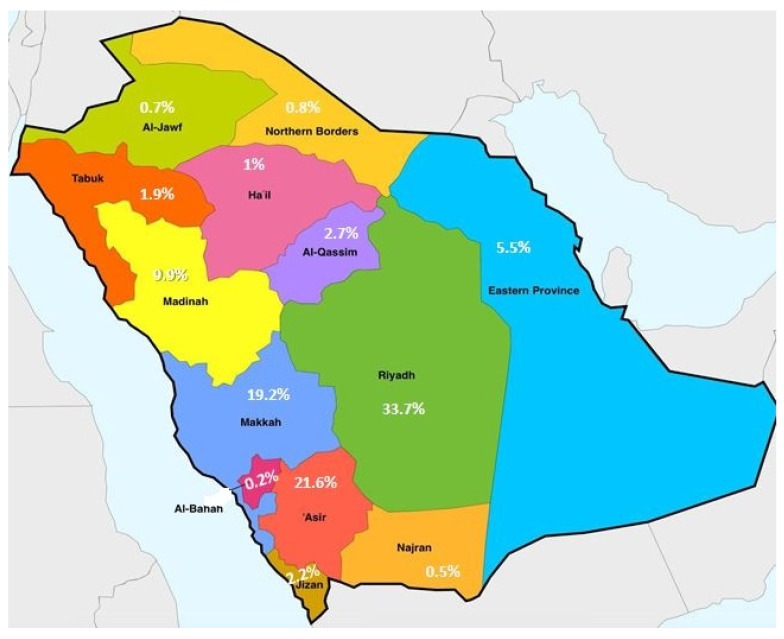
Distribution of participants based on the region in Saudi Arabia.

**Figure 2:**
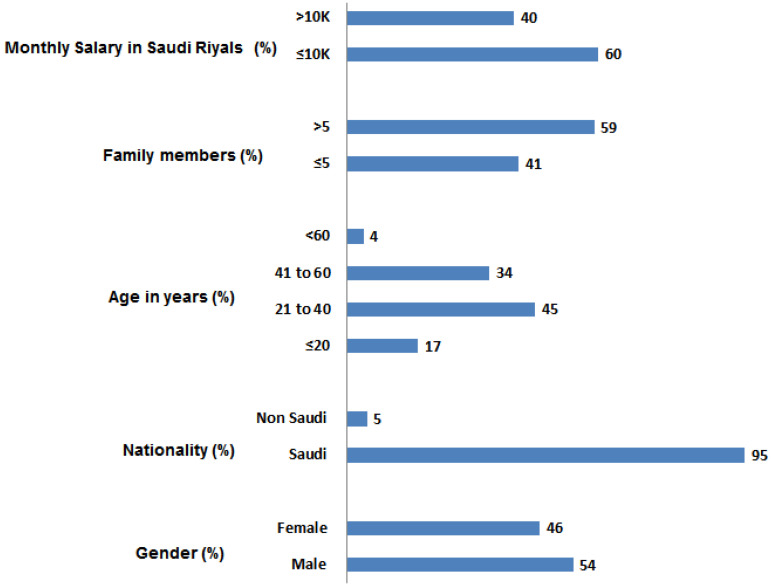
Demographic data of population participated in the study.

**Figure 3:**
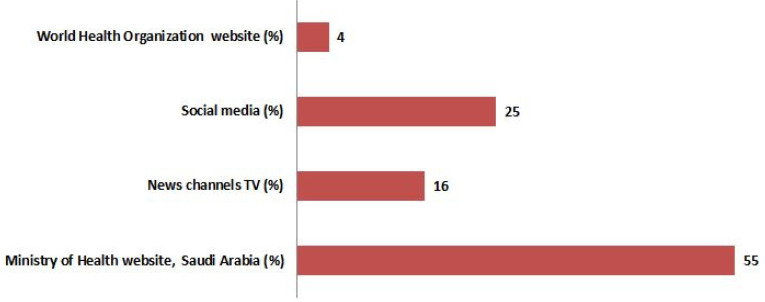
Details of the source of information utilized by the Saudi Arabian population participated in the study.

A total of 2013 participants responded in the study from various regions of Saudi Arabia (Figure 1). The majority of the participants were from the Riyadh region (33.7%), followed by the Asir region (21.5%). Amongst the participants’ males were 1088 (54%), and 925 (46%) were females. The distribution of study participants was shown in Figure 1. The majority (95%) of the total participants was Saudis by nationality, and 59% of the participants confirmed that they had more than five members in their family, and 60% had a monthly income of less than 10 K. The age-wise distribution of participants was ≤20 years (17%), 21–40 years (46%), 41–60 years (34%), and >60 years (3%), respectively. All the demographic characteristics were summarized in Figure 2. The Saudi Arabian population utilized various sources for information on COVID-19, that include the MOH website, Saudi Arabia (55%), social media (24%), news channels (16%), and WHO (4%) see Figure 3.

The mean percentage of positive answers of perception, preventive practices, and oral health practices percentage of achieved scores were summarized in Table 2 based on the study population’s demographic characteristics. Amongst the participants’ females (70%), Saudis (69%), 41–60 years age group (66%), ≤5 family members (71%), and ≤ 10 K SAR salary (70%) showed higher mean percentages for perceptions on COVID-19. For preventive practices, females (78%), Saudis (75%), 41–60 years age group (70%), >5 family members (75%), and >10 K SAR salary (75%) achieved a higher mean percentage. While females (26%), Saudis (32%), 41–60 years age group (35%), ≤5 family members (50%), and ≤10 K SAR salary (33%) mean percentages achieved for oral health care perception among population live in Saudi Arabia (Table 2).

The present study results showed that approximately 89.5% of the participants had proper awareness about COVID-19 and its symptoms (Table 3). Understanding the COVID-19 symptoms was significantly less among the non-Saudi participants than the Saudi participants (*p* = 0.000). Awareness on COVID-19 was found to be more in females, those below 60 years of age, having less than five family members, and monthly income of more than 10 K (*p* > 0.05). Amongst the participants’ females (90%), Saudis (90%), 41–60 years age group (91%), ≤5 family members (91%), and >10 K SAR salary (91%). There was a statistically significant difference evident in the comparison of nationality and monthly salary. The majority of the males (35%), non-Saudis (55%), and participants belongs to 21–40 years age group (39%), and ≤ 10 SAR monthly salary opined that monthly income is going to affect in lockdown period and the statistically significant was evident (*p* > 0.05). Regarding the financial consumption rate during the lockdown, 55% of the females and 54% of ≤ 10 K SAR monthly salary participants stated that the financial consumption rate would affect (*p* < 0.05). The nationality, various age groups, and the family members’ number showed no statistical difference (*p* > 0.05).

The majority of the participants were willing to disclose to hospital authorities if they have suspicious symptoms of COVID-19. Amongst them are males (95%), Saudis (94%), 41–60 years age group (96%), ≤5 family members (95%), and >10 K SAR salary (95%) willing to disclose to the hospital authorities. All the comparisons showed statistically significant (*p* < 0.05). Mixed views were observed regarding preventive practices (wearing a facemask, social distancing, washing hands with soap, and using sanitizer) among the Saudi Arabian population during pandemic based on gender, nationality, age groups, number of family members, and monthly income (Table 4). On the other hand, regarding following the curfew rules, females (69%) than males (54%), Saudis (61%) than non-Saudis (57%), age group belongs to more than 60% (71%) than other age groups committed to the restrictions. There was a statistical difference evident among these comparisons (*p* > 0.05). Regarding refusing family visitors during the lockdown period, two-thirds of females and participants belong to the 41–60 years age group (*p* < 0.05). Regarding the study participants’ oral health perceptions, overall, significantly fewer people experienced dental pain or dental discomfort during the lockdown period (Table 5). Amongst, the majority of them were females (37%), non-Saudis (30%), <20 years (38%), and ≤10 K SAR (32%), the findings were statistically significant (*p* < 0.05). Significantly a smaller number of the participants were willing to visit the dentist during the lockdown period, which includes 17% of males (*p* > 0.05), 16% of Saudis (*p* < 0.05), 19% of ≤20 years age group (*p* > 0.05), 35% of them having more than five family members (*p* < 0.05) and 19% ≤ 10 K SAR (*p* < 0.05).

## 4. Discussion

Perceptions, preventive practices, and oral health practices among the Saudi Arabian population regarding the COVID-19 pandemic were assessed in the present study. This study is the first to discuss all these aspects among the Arabian population residing in various Saudi Arabia regions to the best of the authors’ knowledge. In the present study, the overall awareness of COVID-19 was 89.5% of the study population. A similar observation was observed in the Cameroon population [19] found a similar score (84.19%). Contrarily, our findings did not agree with the African-based population study [3], where the authors found 73.5% of awareness regarding COVID-19 in their study population. Earlier, a similar survey in Saudi Arabian population was published in which the knowledge score was found to be 81.64%, which is less than the present study suggesting that the people of Saudi Arabian have a better understanding of the present scenario and are updating them with the COVID-19 knowledge [20]. Overall, 90% of the participants discerned that the incidence of COVID-19 could be minimized by staying at home and not meeting with others in public places during the lockdown period. This knowledge was significantly more in females than in males (*p* = 0.000). In addition, 89% of them knew about the symptoms of COVID-19. In a similar study by Honarvar et al. [21] in the Iranian population, it was found that only 4.8% of the participants were not aware of the symptoms of COVID-19, which suggests that the people of Saudi Arabia are more knowledgeable about the current global pandemic.

The overall perception in the present study was found to be 56.5%. A similar bi-national survey from the African population [3] observed 64% regarding COVID-19 among the study population. Thirty-six percent of the present study participants thought their monthly income would be affected during the lockdown period, and 61% monitor the daily new cases of affected people by COVID-19 in their city. Eighty-four percent recommend their family members and neighbors to use face masks and gloves for safety when they go out during the lockdown period, and 50% thought that the financial consumption rate would be decreased during the lockdown period. In the present study, perceptions were found more in females (*p* = 0.000), non-Saudi participants (*p* = 0.000), those below 60 years of age, those with more than five family members, and those having an income of less than 10 K (*p* = 0.002).

The overall preventive practice score in the present study was 74.3%. A practice score of 60.8% was observed in a similar study by Ngwewondo et al. [19] in the Cameroon population. In the present study, 93% of the participants agreed that if Coronavirus symptoms exist, they would disclose it and go to the hospital for screening (*p* = 0.000); 61% accepted that they feel an embarrassment in non-shaking hands with others because of the customs and traditions during this COVID-19 lockdown period, whereas 21.8% did not feel any embarrassment in non-shaking hands, in which females were significantly more in number than males (*p* ≤ 0.001). In addition, 89.5% use a face mask and wash 1 s with soap and water or sanitizer to prevent Coronavirus transmission (*p* ≤ 0.001). Sixty-one percent of the participants rated their commitment as 5, suggesting a total commitment to lockdown periods and curfew laws. 62.6% refused their family visitors during the COVID-19 lockdown period, and 79% maintained social distance (*p* ≤ 0.001).

The overall oral health care perceptions were 60% in the present study. 74% of the participants did not experience any dental pain or discomfort during this COVID-19 period, whereas 26% had felt dental pain or discomfort during this COVID-19 period. 67.4% did not prefer to visit the dentist personally during this COVID-19 period, and 52% would like to call the dentist explaining their dental problems rather than visiting the dentist personally before treatment. In the present study, females were found to be more knowledgeable than males. Also, perception, preventive practice, and oral health care perception were more in females than males. Hence, more knowledge was associated with increased perception, more preventive practice, and oral health care perception in females in the present study. Honarvar et al. [21] studied the perception of COVID-19 among the Iranian population and found increased knowledge and preventive practice in females. Similar observations were made by Al-Hanawi et al. [20], Brug et al. [22], Bish and Michie [23]. In contrast, Ngwewondo et al. [19] have observed more preventive practice in males than females. Saudi participants were more knowledgeable than the non-Saudi participants in the present study (*p* > 0.05); nonetheless, perceptions were less among the Saudi participants than the non-Saudi participants. However, preventive practice and oral health care perception were more in Saudi participants than in the non-Saudi participants (*p* < 0.05). Hence, their increased knowledge was associated with higher preventive practice and oral health care perception. The perception was more in participants below 60 years of age. However, preventive practice and oral health care perception were found more in elderly participants over 60 years because older people are more prone to infectious diseases. The present study also observed more preventive practices in older people, and these findings are consistent with a Chinese study [18] and a Nigerian study [24] Zhang et al. [18]. This outcome explains that older people with or without comorbid diseases gained more preventive practices than other age groups. Similarly, increased age associated with an increased preventive approach was observed by Zhong et al. [18], Al-Hanawi et al. [20], and Lorfa et al. [24] in their respective studies. Honarvar et al. [22] have observed less knowledge and less preventive practice in the elderly age groups. Participants with less than five members were found to have more knowledge and oral health care perception, but the perception and preventive practice were found more in those with more than five family members. These differences were not statistically significant. None of the reported studies have reported family members’ effect on perception and preventive practices regarding COVID-19 and oral health care perceptions during COVID-19. Perceptions, preventive practice, and oral health care perception were observed more in participants with a monthly income of more than 10 K, whereas perception was observed more in those with less than 10 K income (*p* = 0.000). Al-Hanawi et al. [20] have also found more awareness of COVID-19 in participants with higher income in their study.

The COVID-19 pandemic outbreak severely impacted the healthcare profession, especially in dentistry [6,8]. This pandemic has changed dental care providers’ opinions and opinions of dental care receivers [25,26,27]. Almost 50% of participants from an American study reported delaying their dental appointments due to the COVID-19 pandemic [28]. Only a few participants preferred to visit the dental operatory. Comparing the gender, nationality, monthly income, and the number of family members showed statistical significance. However, no prior study compared these factors on dental visit preference during COVID-19 lockdown from Saudi Arabia. Comparatively, most survey participants preferred to have a telephonic conversation with the dentist before a dental appointment. The health authority provides guidelines for safe and effective dental practice during this pandemic outbreak [29]. The use of personal protection equipment (PPE), including N95 respirators, face shields, eye protection, surgical masks, and protective clothing, is strictly recommended to avoid the contraction of COVID-19 in the dental operatory. It explains the need for a telemedicine model in such pandemic situations. A recent article by Benzian and Niederman [26] explained SAFER dentistry that could benefit both the patients and dental care providers. Focusing on the source of information regarding COVID-19 amongst the people is also plays an essential role in perception and preventive practices. In the present study, only 4% of the participants relied on the WHO website. In Alanazei et al. [30] study, 18% of the participants preferred the WHO website. However, Alanazei et al. [20] findings are not comparable because they used multiple options for the source of information utilized for COVID-19. It has also been reported that the different sources of information had copious associations with the assurance in managing with concern to COVID-19 [31]. Participants in the present study used a multiplicity of sources for information concerning the COVID-19. The Saudi Arabia Ministry of Health website was utilized by most participants (55%) in the study to know information on COVID-19. Alanezi et al. [30] also reported similar findings. The authors reported that 65% of the participants utilized the Ministry of Health, Saudi Arabia, as a source of information. Furthermore, it explains the health authorities from Saudi Arabia were very successful in reaching people in the country with information regarding COVID-19 based on our study. Risk perceptions refer to people’s spontaneous estimations of vulnerabilities they might be exposed to, with unwanted effects that the population associates with a precise cause [14,32,33]. Risk perception of a country means interpretations of the populations. Sharma et al. [34] used a fourth-generation multi-theory model (MTM) to explain and explore the hand-washing behavior among American college students.

A survey from Saudi Arabia also confirmed the risk perceptions regarding COVID-19 among dental undergraduate students [35]. A multinational study from 15 countries [36] reported that there need to develop a proper public health intervention to address college students’ emotional and psychosocial needs during this COVID-19 Pandemic. A recent study [37] reported that dental specialists showed adequate knowledge regarding preventive measures. Furthermore, a recent study [38] suggested that it is imperative to promote the infection control protocols among dental students through training programs to avoid the potential risk of COVID-19. Cori et al. [39] opined that government authorities’ administration of risk communication is required to establish consciousness and rationality. A recent study [40] found a higher prevalence rate of anxiety, depression, sleep problems, stress, and psychological distress among the general population during this pandemic outbreak. However, in the present study, we have not evaluated the psychological aspects of the populations. There is a need to evaluate the stress levels among the general population in Saudi Arabia. Based on the present study findings, the authors opine that the perception of risk regarding COVID-19 might associate with perceptions about COVID-19 and that will impact preventive behavior.

### Limitations

A diversity of variables could predict the general population’s reactions to the COVID-19 pandemic to avoid infection. The present study aimed to establish Saudi Arabian residents’ perceptions, preventive practices regarding CVOID-19, and oral health practices during COVID-19. The present study utilized a limited number (2013) of participants from Saudi Arabian from various provinces, and studies with a larger sample size are recommended. The population from illiterate and unprivileged groups did not participate in the study. Therefore, the findings from these segments of the society were not gathered in the present study. A total of 2013 people participated from various Saudi Arabia regions, and the majority were from the Riyadh region (33.7%), followed by the Asir region (21.5%). The region-based analysis was not done in the present study. There was a comparatively remarkable difference among participants from the various areas. However, this could also be a limitation of the study. To the best of our knowledge, the present study qualifies as the first reported study from Saudi Arabia to the best of our knowledge assessed the perception, preventive practices, and oral health practices among the population in Saudi Arabia with concern to COVID-19. Other limitations include study design, self-administered questionnaire, convenience sampling, which cannot be generalized to the study findings. There was no equal distribution of the participants from various provinces of Saudi Arabia, and the majority of the study participants were from Riyadh and Asir. Nonetheless, the findings in the present study possibly will be used as a reference to explore variance with sample size made up of a population without internet access.

## 5. Conclusions

The present study concludes that although the knowledge is sufficient amongst the Saudi Arabian population, there is a need to improve participants’ perception, preventive practice, and oral health care perceptions to improve their active involvement in controlling the COVID-19 transmission. The Ministry of Health Saudi Arabia website is the most reliable source of information COVID-19. The present study suggests that the Ministry of Health in Saudi Arabia successfully created awareness, and mixed responses were observed on preventive practices among the Saudi Arabian population. Comprehensive details of COVID-19 perceptions, preventive practices, and oral health care practices among the Saudi Arabian population based on gender, nationality, age groups, family members, and monthly incomes were discussed in this study.

## Figures and Tables

**Table 1:** Questions related to perception (P), preventive practice (PRA), and oral health care perceptions (D).

No.	Questions
P1	Do you think the Corona virus incidence can be reduced by staying at home and not meeting with others during lockdown?
P2	Do you know the symptoms of the Coronavirus?
P3	Do you think your monthly income is going to effect during this lockdown period?
P4	Do you monitor the daily new cases of affected people by Coronavirus in your city during the lockdown period?
P5	Do you recommend your family members and neighbors use face masks and gloves for safety when they go out during this lockdown period?
P6	How do you think the financial consumption rate will be affected during this lockdown period?
PRA1	If symptoms of Coronavirus exist, would you disclose and go to the hospital for screening?
PRA2	Do you feel an embarrassment in others’ non-shaking hands because of the customs and traditions during this COVID-19 lockdown period?
PRA3	Are you using a face mask and washing hands with soap and water or sanitizer to prevent Coronavirus transmission in lockdown period?
PRA4	To what extent do you commit to lockdown period and curfew laws?
PRA5	Are you able to refuse your family visitors during the COVID-19 lockdown period?
PRA6	Are you maintaining social distance?
D1	Have you felt any dental pain or discomfort during this COVID-19 period?
D2	Do you prefer to visit the dentist personally during this COVID-19 period?
D3	Are you happy to make a call with a dentist explaining your dental problems rather than visiting the dentist personally before treatment?

**Table 2:** Overall mean percentage scores of perception, preventive practice, and oral health care perceptions.

Parameters		Perception (P)	Preventive Practice (PRA)	Oral Health Care Perception (D)
Gender	Female	70%	78%	26%
	Male	67%	71%	21%
Nationality	Non Saudi	68%	72%	29%
	Saudi	69%	75%	32%
Age	<20 year	59%	67%	35%
	21–40	65%	67%	31%
	41–60	66%	70%	31%
	>60 year	65%	67%	26%
Family member	1–5	71%	74%	50%
	>5	67%	75%	48%
Income	<	70%	74%	33%
	>	68%	75%	31%

**Table 3:** Comparison of the effect of demographic factors on perception score during covid-19 in Arabian population.

Questions	Demographic Factor																
	Gender			Nationality			Age (in years)					Family Members			Income (SAR)		
	F	M	*p* Value	NS	S	*p* Value	<20	21–40	41–60	>60	*p* Value	<5	>5	*p* Value	<10 K	>10 K	*p* Value
P1	93%	90%	0.03 *	82%	92%	<0.001 *	92%	90%	93%	87%	0.20	93%	90%	0.05	90%	92%	0.09
P2	90%	89%	0.80	87%	90%	0.03 *	87%	89%	91%	85%	0.29	91%	88%	0.11	87%	91%	<0.001 *
P3	31%	35%	<0.001 *	55%	32%	<0.001 *	21%	39%	32%	34%	<0.001 *	34%	34%	0.08	41%	29%	<0.001 *
P4	60%	63%	0.05	52%	62%	<0.001 *	44.%	64%	66%	69%	<0.001 *	65%	59%	0.01 *	58%	64%	<0.001 *
P5	90%	79%	<0.001 *	83%	84%	<0.001 *	85%	84%	85%	81%	0.14	86%	83%	0.14	87%	82%	<0.001 *
P6	55%	48%	0.01 *	46%	52%	0.79	27%	26%	29%	36%	0.13	54%	50%	0.08	54%	50%	0.04 *

Perception Questions—P1, P2, P3 and P4; F = Female; M = Male; NS = None Saudi; S = Saudi; SAR = Saudi riyal; * Significant *p* < 0.05.

**Table 4:** Comparison of effect of demographic factors on preventive practice score during COVID-19 in Arabian population.

Questions	Demographic Factor																
	Gender			Nationality			Age (years)					Family Members			Income (SAR)		
	F	M	*p* Value	NS	S	*p* Value	<20	21–40	41–60	>60	*p* Value	<5	>5	*p* Value	<10 K	>10 K	*p* Value
PRA1	92%	95%	<0.001 *	88%	94%	<0.001 *	89%	93%	96%	91%	<0.001 *	95%	93%	0.01 *	90%	95%	<0.001 *
PRA2	66%	54%	<0.001 *	64%	59%	0.10	20%	27%	19%	10%	<0.001 *	58%	61%	0.48	58%	60%	0.68
PRA3	93%	89%	0.01	84%	91%	0.02 *	92%	92%	90%	83%	<0.001 *	92%	90%	0.16	92%	90%	0.41
PRA4	69%	54%	<0.001 *	57%	61%	<0.001 *	63%	58%	63%	71%	<0.001 *	58%	63%	0.44	62%	60%	0.31
PRA5	65%	60%	0.01	60%	63%	0.78	60%	58%	69%	67%	<0.001 *	63%	62%	0.52	61%	63%	0.13
PRA6	83%	75%	<0.001 *	77%	79%	0.04 *	79%	76%	82%	81%	0.18	78%	80%	0.39	79%	79%	0.21

Preventive practice Questions—P1, P2, P3 and P4; F = Female; M = Male; NS = None Saudi; S = Saudi; SAR = Saudi riyal; * Significant *p* < 0.05.

**Table 5:** Comparison of the effect of demographic factors on oral health care perception score.

Questions	Demographic Factors																
	Gender			Nationality			Age (in Years)					Family Members			Income (SAR)		
	F	M	*p* Value	NS	S	*p* Value	<20	21–40	41–60	>60	*p* Value	<5	>5	*p* Value	<10 K	>10 K	*p* Value
D1	37%	19%	<0.001 *	30%	27%	0.62	38%	28%	21%	9%	<0.001 *	27%	27%	0.11	32%	24%	<0.001 *
D2	15%	17%	0.37	9%	16%	<0.001 *	19%	18%	13%	13%	0.07	14%	18%	0.01 *	19%	14%	<0.001 *
D3	52%	52%	0.98	47%	53%	0.40	49%	48%	58%	56%	<0.001 *	53%	52%	0.01 *	49%	54%	<0.001 *

Oral health care perception questions—D1, D2, and D3; F = Female; M = Male; NS = None Saudi; S = Saudi; SAR = Saudi riyal; * Significant *p* < 0.05.

## Data Availability

The data that support the findings of this study are available from the corresponding authors upon reasonable request.
